# Understanding and Reporting PFAS Exposure: Wide-Scope Retrospective Suspect Screening of High-Resolution Mass Spectrometry Data from Exposomics Studies

**DOI:** 10.1021/acs.est.6c03864

**Published:** 2026-07-22

**Authors:** Lapo Renai, Federica Calabró, Viktoriia Turkina, Pradeep Dewapriya, Kevin V. Thomas, Stefano Papazian, Saer Samanipour

**Affiliations:** † Van’t Hoff Institute for Molecular Sciences (HIMS), 84709 University of Amsterdam, Amsterdam 1090 GD, The Netherlands; ‡ 1974 Queensland Alliance for Environmental Health Sciences (QAEHS), 20 Cornwall Street, Woolloongabba, Queensland 4102, Australia; § Department of Environmental Science, 7675 Stockholm University, Stockholm 106 91, Sweden; ∥ National Facility for Exposomics, Science for Life Laboratory, Stockholm University, Solna 171 65, Sweden; ⊥ UvA Data Science Center, University of Amsterdam, Amsterdam 1000 GG, The Netherlands

**Keywords:** nontargeted analysis, reanalysis, human biofluids, perfluorochemicals, human biomonitoring, continuous reporting

## Abstract

Per- and polyfluoroalkyl substances (PFAS) comprise thousands of persistent and structurally diverse chemicals that contribute to chronic human exposure. Yet, routine biomonitoring targets only a limited set of regulated legacy compounds, underestimating overall exposure. We present a retrospective, wide-scope suspect screening analysis framework to extract PFAS information from archived LC–HRMS exposomics datasets. A curated list of 1,666 PFAS, filtered for reversed-phase LC compatibility and enriched with structural descriptors, predicted retention indices, and MS^2^ fragments, was used to reprocess two human biomonitoring studies involving firefighter serum pools and population-based plasma samples. Candidate features were prioritized using precursor accuracy, isotopic patterns, and MS^2^ similarity, followed by spectral quality filtering and retention time-index regression models based on isotopically labeled standards, reinforcing identification confidence. The reanalysis recovered up to 80% of previously reported PFAS while revealing overlooked high-confidence carboxylic, sulfonic, and sulfonamidoacetic acid homologues, and >30 tentative PFAS series, encompassing isomeric clusters (alcohols and ethers) and structurally distinct subclasses (alkylpyrimidine PFAS). The reanalysis framework also revealed shared homologue patterns across exposure contexts. By framing PFAS chemical space and providing a reusable repository of confirmed and tentative structures, this framework reinterprets HRMS biobanked data, improving PFAS pattern assessment.

## Graphical abstract

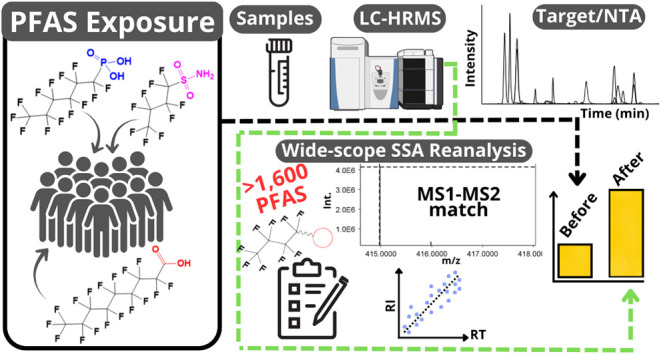

## Introduction

Per- and polyfluoroalkyl substances (PFAS) are a group of anthropogenic chemicals defined as “any chemical with at least a perfluorinated methyl group (−CF_3_) or a perfluorinated methylene group (−CF_2_−)”.^1^ PFAS’s predominant molecular scaffold consists of a hydrophobic alkyl chain and a major terminal hydrophilic group. Variations in fluorination degree, chain length, and polar substituents generate an extensive structural and physicochemical diversity among PFAS. Widespread use of PFAS in both direct applications (e.g., packaging, textiles, and fire-fighting foams) and indirect contamination pathways (e.g., releases from wastewater treatment plants) has led to their global environmental diffusion.^2^ Actions have been undertaken by worldwide authorities and policymakers to monitor and manage human exposure to PFAS, including prohibitions and restrictions on the use of specific perfluorinated chemicals (e.g., perfluorooctanesulfonic acid and its derivatives, C6–C14 perfluorocarboxylic acids),^3^ which, however, led to the exponential production and use of short-chain and new-generation PFAS as alternatives.^4,5^ For these reasons, the current PFAS chemical space that the environment and humans are exposed to contains over 4600 registered-for-production and regulatory-informed structures.^6–9^ Despite this, environmental exposure assessments rely predominantly on targeted analytical workflows that typically quantify a “regulatory” restricted panel of PFAS, largely dominated by perfluoroalkyl carboxylic acids (PFACAs) and perfluoroalkyl sulfonic acids (PFASAs). This analytical bias is often driven by analytical standard availability, and it is consistently observed across environmental and human biomonitoring studies, where these legacy compounds account for the majority of detected and quantified PFAS; structurally diverse or emerging PFAS subclasses are only marginally reported or remain undetected.^10–12^ This highlights a critical gap between the known PFAS chemical space and the fraction effectively captured by conventional biomonitoring-targeted methodologies.

Nontarget analysis (NTA) workflows coupled to liquid chromatography high-resolution mass spectrometry (LC–HRMS) have significantly advanced our ability to characterize human exposure to the complex PFAS universe through high-resolution mass records of precursors and fragments.^13–15^ Importantly, because HRMS data archive the complete sample chemical information, the acquired datasets enable the retrospective analysis supported by improved and growing suspect lists (e.g., wide-scope experimental or in silico MS-based lists)^16–18^ or newly retrospective discovery approaches.^19^ Reprocessing biobanked datasets could recover the overlooked chemical information to construct a more complete picture of human PFAS exposure, including novel or transformation-related compounds, as well as (organo)fluorine mass balance.^6,20^ In this context, NTA tools such as FluoroMatch and ΔPFAS leverage diagnostic mass defects, recursive fragmentation, and neutral loss patterns to capture homologous series and structurally related PFAS, thereby enabling a broader exploration of the PFAS chemical landscape beyond conventional targeted biomonitoring that is limited by analytical standard availability.^21–24^ Despite these advantages, identification strategies primarily based on canonical structural motifs or characteristic losses (e.g., ΔCF_2_, ΔCF_3_, ΔHF), often derived from common or legacy analytical standards, may not fully represent the diversity of the PFAS chemical space. While highly effective for recognizing perfluoroalkyl homologous patterns, such approaches can underrepresent structurally distinct subclasses, including fluorotelomer-based, ether, and polyether PFAS.^25^ In addition, the absence of a structure–chromatographic coherence layer—linking molecular scaffolds to expected retention behavior—limits the ability to evaluate whether detected features behave consistently with their proposed structures under the applied LC conditions. As a result, candidate annotations may remain chemically plausible but insufficiently constrained within the method-specific analytical space. That is why cause–and–exposure relationships are predominantly evaluated using “easy-to-detect” PFAS prioritized by regulatory initiatives, such as PFACA and PFASA homologs.^26–28^ Suspect screening analysis (SSA) provides a complementary framework by incorporating compound-specific information such as exact mass, molecular formula, isotopic ratio, and curated MS^2^ fragments to prioritize structurally plausible PFAS, alleviating the need for reference standards.^29,30^ Compared to the aforementioned data-driven NTA approaches, SSA improves interpretability by anchoring candidate selection within curated chemical knowledge. Nevertheless, without evaluating whether a detected candidate exhibits chromatographic behavior consistent with its proposed PFAS structure under the applied LC conditions, identification may remain chemically plausible but insufficiently contextualized within the method domain. Retention information could be, in principle, integrated within SSA as a structure–chromatographic coherence filter, where correlation between observed retention times and structure-predicted retention indices enables the exclusion of candidates showing an inconsistent structure–chromatographic trend, thereby improving identification reliability beyond mass spectral evidence alone.^31^

In response to this gap, we propose a wide-scope retrospective SSA framework designed to expand the evidence on PFAS (overlooked) exposure in archived LC–HRMS exposomics datasets from human monitoring studies. The approach integrates a structurally diverse PFAS suspect list curated from the OECD inventory with MS-based evidence and a structure–chromatographic coherence filtering step based on retention time–retention index (RT–RI) relationship. By combining broad-coverage fragmentation-informed screening with retention behavior constraints, this framework aims to both broaden PFAS coverage beyond legacy compounds and improve the plausibility of tentative identifications within the analytical method domain. Importantly, this approach enables the systematic evaluation of whether structurally diverse and previously underrepresented PFAS subclasses can be captured in retrospective HRMS datasets, thereby addressing a key limitation of existing NTA workflows. To demonstrate its applicability, we reanalyzed two human biomonitoring datasets and assessed the extent to which expanded suspect screening and structure-informed filtering improve PFAS detection and interpretation across different exposure scenarios. To support our hypothesis about the broader implications of PFAS exposure for human health, confirmed and tentative structures are collected in an open repository to expand exposure knowledge for the community.

## Materials and Methods

### Retrospective Framework

The wide-scope SSA strategy was integrated to support and enhance understanding of PFAS exposure by systematically reanalyzing LC–HRMS datasets from human studies (Figure 1). Raw data were converted to .*mzXML* format to extract MS^1^ and MS^2^ information. A suspect list of over 1,600 PFASs was curated from native analytical standard data and the OECD PFAS inventory, filtered for reversed-phase LC (RPLC) compatibility, and enriched with structural (e.g., InChIKeys, SMILES, and exact mass) and method-related (e.g., ionization mode and retention index) descriptors, MS^2^ fragments, and identification confidence levels. Suspect screening across the LC–HRMS data identifies preliminary matches based on defined precursor accuracy, isotope pattern, and fragment confirmation (i.e., MatchFactor), generating a “raw” candidate list. A data-based filtering strategy combining spectral similarity, MS^1^-MS^2^ signal intensity (most intense and coherent), and confidence ranking (highest level among duplicates) minimizes redundancies, while a retention time–retention index (RT–RI) regression model fitting further refines identifications according to chromatographic behavior. The final curated list provides reanalyzed and reportable PFAS identifications with confidence levels ≤ 2 (*leq2*) and ≥ 3 (*geq3*), enabling improved evaluation of human exposure.

**Figure 1:**
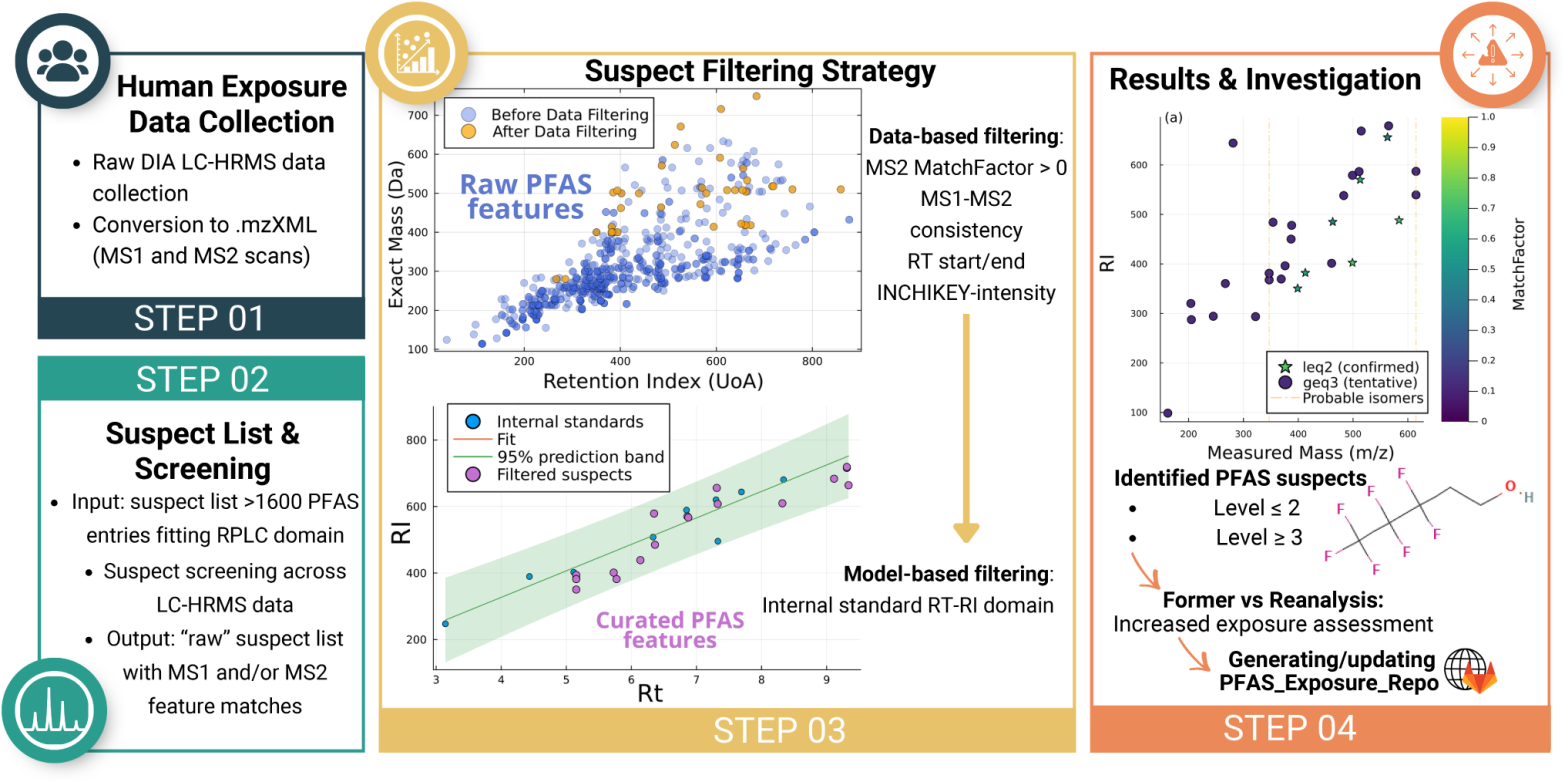
Overview of the retrospective SSA framework for PFAS detection in LC–HRMS exposomics datasets. Step 01: collection of raw DIA LC–HRMS data and conversion to .*mzXML* format, including MS^1^-MS^2^ scans. Step 02: wide-scope suspect screening using a curated PFAS list (1,666 entries) to generate a raw list of candidate features based on MS^1^ and/or MS^2^ matches. Step 03: multistep filtering strategy combining data-driven criteria (e.g., MatchFactor > 0, MS^1^-MS^2^ consistency, retention time constraints) and model-based filtering using RT–RI relationships derived from internal standards, resulting in a curated set of analytically coherent PFAS features. Step 04: results interpretation and exposure assessment, including classification by confidence level (*leq2* and *geq3*), comparison with previous studies, and integration into a PFAS exposure repository.

### Data Collection

Two LC–HRMS datasets, acquired in negative ionization mode, were reanalyzed using the proposed SSA-based workflow. The first dataset (“Dataset 1”) consists of serum pools from the general Australian population and PFAS-exposed firefighters, including *n* = 100 and *n* = 7 individuals, respectively.^32^ The second one (”Dataset 2”) involved *n* = 161 biobanked plasma samples from the Northern Sweden Health and Disease Study (NSHDS), including 100 breast cancer–free women.^33^ Both studies analyzed the samples using reversed-phase LC–HRMS under broadly selective, non-targeted conditions, combining full-scan MS^1^ with MS^2^ data-independent acquisition (DIA, Table S1). DIA employed predefined fixed-width isolation windows (Dataset 1 = 50 Da, Dataset 2 = 237 Da) for acquisition-specific precursor mass ranges and window overlaps (Dataset 1 = 1 Da, Dataset 2 = 10 Da). Study samples were spiked with isotopically labeled internal standards to ensure data quality and monitor chromatographic and ionization stability.

### Wide-Scope Suspect Screening Analysis

#### Curated PFAS Suspect List

The complete list of the OECD structures defined as PFAS and present in the market (≃4900)^34^ was used as the starting point for setting up a wide-scope PFAS list. For each listed entry, structural metadata were automatically retrieved from PubChem (*PubChemCrawler.jl* package),^35^ namely SMILES, InChIKey, name, formula, and monoisotopic exact mass. Since missing from the OECD list, MS^2^ components were retrieved from experimental data generated by state-of-the-art PFAS MS libraries, yielding a list of the 63 most frequently detected fragments assigned to each OECD entry. The OECD list was later restricted (*n* = 1,627) to PFAS entries with available physicochemical descriptors in PubChem (i.e., XLogP, TPSA, and H-bond/donor counts via *PubChemCrawler.jl*; ≃3,200) fitting RPLC space based on their predicted Retention Indices (100 < RIs < 1000, UoA scale)^36^ and their classification as “inside” the RPLC chemical space (applicability domain ≤ 1) using the model by van Herwerden et al.^37^ (Figure S1 of the Supporting Information). In addition, experimental PFAS suspect entries were retrieved from in-house recorded LC–HRMS data of PFAS standard mixtures and related literature.^24,38–40^ PFAS entries were further labeled according to identification confidence levels.^41^ All suspects supported by experimentally derived MS^2^ data (i.e., confidence levels 1 and 2) were classified as level ≤ 2 (*leq2*, *n* = 39 out of 1,666 entries). The remaining entries (*n* = 1,627) lacked direct experimental MS^2^ evidence and were therefore assigned to level ≥ 3 (*geq3*), relying on a set of 63 representative fragments derived from available PFAS in-house MS library (*n* = 414 known PFAS).^42^ The compiled suspect list (*n* = 1,666 entries in total) is available in the Supplementary_Tables folder at https://doi.org/10.6084/m9.figshare.31182265.

#### Data Import and Suspect Screening

LC–HRMS raw data from the selected studies were converted into .*mzXML* format using MSConvert,^43^ retrieving MS^1^ and MS^2^ scan levels. The .*mzXML* files were imported using the *MS_Import.jl* package,^44^ using an *m*/*z* range of 70–1200 Da and an intensity threshold of 2000 for Dataset 1 and of 10,000 for Dataset 2, as a function of the relative signal background of the mass spectrometers used (i.e., Q-TOF and Orbitrap, Table S1). Suspect screening was carried out on LC–HRMS data using the *Screening.jl* module available in the ULSA package,^45^ modified to retain the RI and confidence level labels in the “raw” suspect output. Suspect screening input parameters were adjusted according to the available datasets, setting a mass tolerance of 5 mDa, a maximum number of isotopic peaks of 5, and a peak width of 0.5. Each LC–HRMS dataset was treated separately. Suspect screening was performed by extracting candidate precursor features from MS^1^ data and associating them with MS^2^ information within a retention time window centered on the chromatographic peak apex (see Supporting information, Section S2 and Figure S2). Such a combination of MS^1^, MS^2^, and chromatographic evidence to support candidate prioritization represents a consistency criterion for DIA data.

#### Data- and Model-Based Filtering

The “raw” suspect lists were further processed by the following curated filtering workflow. First, a data-based filtering step was applied, retaining only suspect entries that met the following criteria: (i) a positive MatchFactor (MatchFactor > 0), indicating weighted agreement between expected and observed fragment patterns; (ii) a consistent intensity relationship between MS^2^ and MS^1^ (MS^2^ < MS^1^); (iii) chemically plausible sulfur-based MS^2^ fingerprints by removing features with characteristic sulfonic-sulfate fragments (m/z 79.9574, 80.9652, 77.9655, 82.9609, 98.9558, 94.9808) when sulfur was absent from the molecular formula; (iv) elution within the chromatographic gradient (i.e., between dead volume and re-equilibration); and (v) the highest MS^1^ intensity and confidence level (*leq2* preferred over *geq3*) for each non-unique PFAS InChIKey or exact mass. This step allows for removing features carrying no structural information (i.e., MS^2^), illogical entries, and redundant entries between confidence levels. Then a PFAS suspect was filtered according to an RT–RI (linear) regression model built on the spiked isotopically labeled internal standards, retaining only the suspect entries falling inside the 95% of the model confidence interval. This ensures that PFAS candidates with an illogical RT–RI relationship are removed (i.e., truly fitting the chromatographic domain), increasing the accuracy of their (tentative) identification. Study isotopically labeled internal standards primarily encompassed perfluoroalkyl carboxylic acids (PFCAs; C4–C14) and perfluoroalkyl sulfonic acids (PFSAs; C4–C8), together with N-ethyl perfluorooctane sulfonamide (N-Et FOSA). Although these compounds predominantly represent legacy PFAS structures, they provide a suitable calibration domain for modeling the relationship between retention time and predicted retention index under the applied RPLC conditions (i.e., hydrophobic interactions).

To integrate the RT–RI model, isotopically labeled internal standard suspect lists (available at Supplementary_Tables—https://doi.org/10.6084/m9.figshare.31182265) were separately created following the procedure reported in the Curated PFAS Suspect List section for each dataset, and suspect screening was carried out only on spiked pooled samples. Because the RT–RI relationship is method-dependent, the regression model should be calibrated using internal standards specific to each analytical setup when applying this workflow to new datasets.

### Retrospective Repository of PFAS Candidates

A retrospective PFAS exposure repository was generated from the filtered candidates across both datasets. The reporting structure and metadata fields were developed in alignment with emerging recommendations for transparent data reporting in NTA studies, thereby facilitating harmonized data sharing and collaborative advancement across the exposomics and environmental chemistry communities.^30^ Each detected PFAS candidate was exported as an individual text-based record with anonymous identifiers assigned sequentially to prevent linkage to personal or sample-specific metadata; no personal identifiers or exposure-level information were retained. Details on its implementation are reported in Section S5 of the Supporting Information. The PFAS repository and pipeline are available at https://gitlab.com/lrenai/PFAS_Exposure_Repo.git.

### Calculations and Data Availability

All calculations were performed on a personal computer (PC) with an Intel Core i9–14900HX central processing unit and 32 GB of RAM operating Windows 11 Education, version 23H2. Data processing and statistical analyses were performed using the Julia language version 1.11.2. The code can be found at https://gitlab.com/lrenai/PFAS_Reanalysis.jl.git. Additional Supporting Information data are available at https://doi.org/10.6084/m9.figshare.31182265.

## Results and Discussion

### Wide-Scope Suspect List Coverage

The use of a wide-scope suspect list, i.e., one that includes a broad array of PFAS entries along with their structural and experimental descriptors, enables not only a broader and more systematic coverage of potential compounds but also standardizes and improves the accuracy and comparability of PFAS identification across studies.^46^ Restricting the OECD list to the “within-RPLC” class retained only structures that are realistically amenable to RPLC–HRMS detection (i.e., Kendrick mass defects > −0.3, 100 < RI < 1000, and 100 < exact mass < 1000). This filtering step effectively removed high molecular weight (e.g., tris­(2-(perfluorododecyl)­ethyl)­phosphate ≃ 2k Da) and poorly ionizable (e.g., perfluoroperhydrobenzyl tetralin, KMD ≃ 0) PFAS that are incompatible with (general) RPLC method conditions typically applied in NTA (Figure S1), being more compatible with alternative analytical strategies, such as gas chromatography-based methods. The final, expanded suspect list comprises 1,666 PFAS that are detectable (i.e., *geq3* tentative confidence level) or have been detected (i.e., *leq2* higher-confidence) in RPLC–HRMS. The curated entries span a broad range of retention indices and cover exact masses from approximately 100 to 900 Da (Figure 2A). The exclusion of approximately 3,200 OECD entries was primarily driven by the absence of the physicochemical descriptors and applicability domain under RPLC conditions. Importantly, for entries classified as *geq3*, predicted RIs will later contribute to complementary, orthogonal evidence, allowing tentative assignments to be supported through a balanced weight-of-evidence approach even when fragmentation agreement is limited. Moreover, the principal component analysis (PCA) of the PFAS chemical space (based on physicochemical descriptors, Figure 2B) demonstrates that the curated suspect list provides substantially broader structural coverage compared to the limited set of PFAS commonly monitored under EU regulation (*n* = 33).

**Figure 2:**
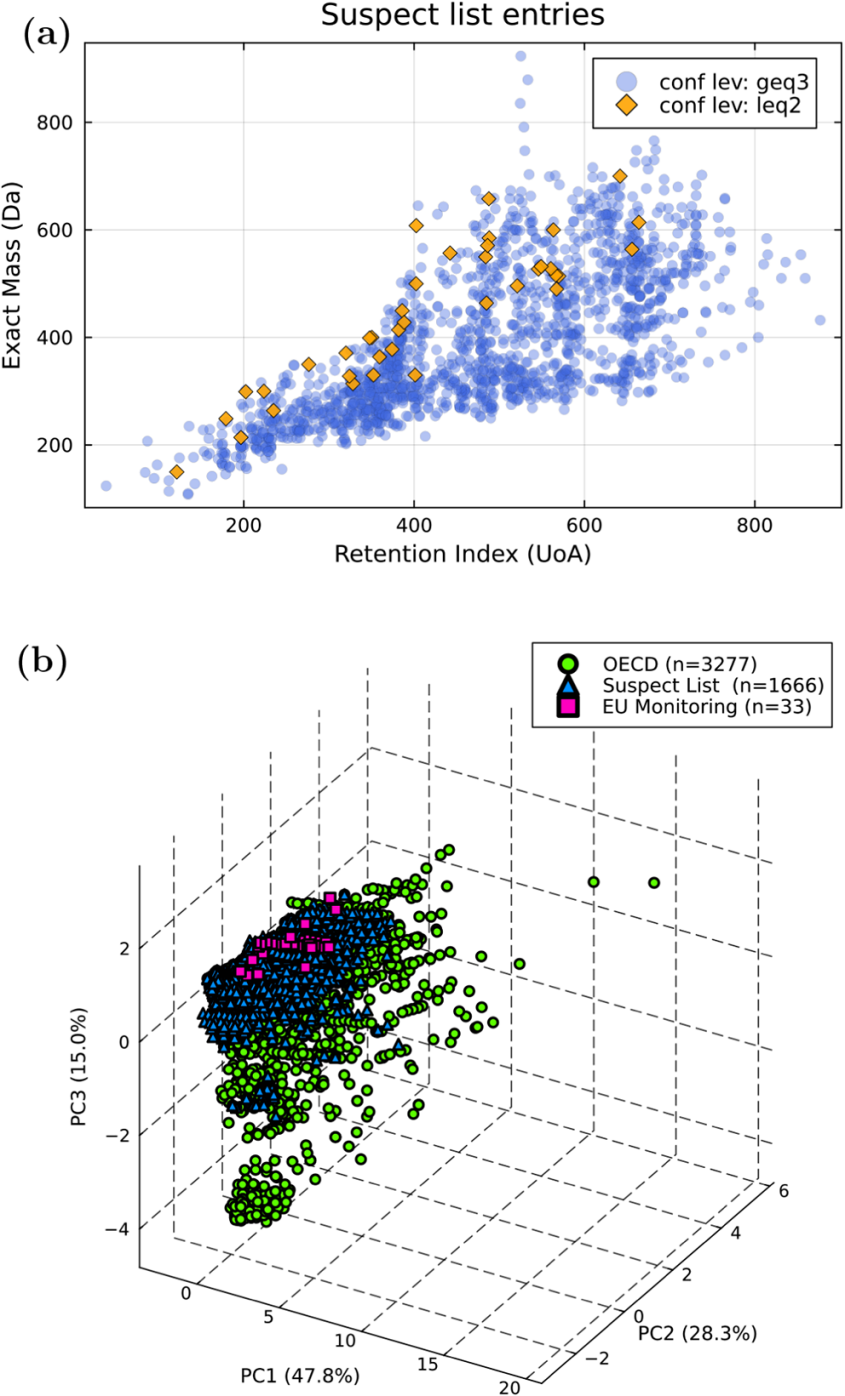
Retention Indices (RI, UoA scale) vs exact mass (Da) distribution of the PFAS entries and related confidence levels included in the curated wide-scope suspect list (a); PFAS chemical space PCA score plot performed on molecular weight, XLogP, TPSA, H-bond/donor counts, and CF-mass defect, with group labels for the OECD-refined PFAS chemical space (green), EU-monitored structures (purple), and the proposed suspect list entries (blue) (b).

### Reanalysis of Exposomics Datasets

The investigated datasets serve as “proof of concept” for interpreting the more complex degree of direct (firefighters) and indirect (background contamination) exposure by PFAS sources. Within the proposed reanalysis framework, both individual-study and cross-study PFAS patterns can be systematically explored, enabling a more comprehensive, yet interpretative, assessment of PFAS exposure. Rather than replacing the original investigations, the framework provides a transparent and consistent retrospective interpretation of archived HRMS data that complements and extends previous findings.

#### Study-Contextualized Filtering Strategy

A central challenge in wide-scope SSA is the transition from a broad set of candidate features to a refined list of confirmed and tentative structures supported by multilevel (MS and retention) analytical evidence. Improving the reliability of tentative identifications by reducing illogical matches enables a more robust interpretation of PFAS occurrence in retrospective analysis. The multistep filtering workflow (Figure S3) implemented here integrates MS-level evidence with chromatographic coherence through an RT–RI linear model, generating a curated PFAS feature set with enhanced annotation reliability. Initial candidate lists (i.e., prefiltering suspect matches) displayed broad distributions in exact mass and retention index space (Dataset 1 = 21,476 candidates; Dataset 2 = 964,761 candidates), containing redundant and low-quality matches that obscure reliable exposure interpretation. The first filtering stage applied data-driven criteria, including MS^2^ agreement on all available fragment information per suspect entry, logical MS^1^–MS^2^ intensity relationships, diagnostic fragment coherence, chromatographic elution window constraints, and consolidation of redundant *m*/*z* and InChIKey assignments (see section Data- and Model-Based Filtering). In this context, MS^2^ agreement was evaluated using a non-null MatchFactor (MatchFactor > 0), reflecting the weighted agreement between expected and observed fragment patterns. The magnitude of this score depends on both the number and relative weighting of fragments available for each suspect entry. For tentative *geq3* entries, fragment matching relies on a curated set of 63 commonly observed PFAS fragments derived from available MS libraries, which represent recurrent substructural motifs rather than compound-specific signatures. As such, fragment agreement is interpreted as a consistency criterion within a weight-of-evidence framework rather than definitive structural confirmation. For instance, a nonzero MatchFactor can also arise from limited fragment agreement (e.g., one or two matches), which does not provide strong structural specificity, a recognized common limitation shared across NTA workflows, including FluoroMatch.^47^ To mitigate this uncertainty, fragment matching is complemented by additional constraints, requiring coelution of precursor and fragment signals within the MS^2^ acquisition window (see Supporting Information, Section S2) and removal of chemically inconsistent matches (i.e., sulfur-containing fragments are disregarded for candidates lacking sulfur in the molecular formula). Together, these constraints reduce the likelihood of false positives while preserving structurally plausible candidates, also mitigating the possible coincidental matches arising from DIA-derived spectra. This multicriteria filtering step substantially reduced the number of candidates for postprocessing (Table S2) while preserving the reliable PFAS chemical space across datasets (Dataset 1 = 66 candidates; Dataset 2 = 1,034 candidates). Subsequently, chromatographic plausibility was evaluated using a linear RT–RI regression model constructed from isotopically labeled PFAS internal standards included in the analytical study design. The internal standards defined a stable RT-to-RI relationship for the chromatographic method (R^2^ = 0.84–0.92 and RMSE = 38.3–56.9 RI units for both datasets, a prediction error of approximately 3–5% over the RI range), allowing prediction of the expected RI for any candidate PFAS based on its experimental RT in the sample PFAS list. It should be noted that the RT–RI models were calibrated using isotopically labeled standards belonging predominantly to legacy PFAS classes (i.e., PFCAs and PFSAs). Consequently, the model should not be interpreted as universally transferable across the entire PFAS chemical space. Nevertheless, under reversed-phase LC conditions, retention is primarily governed by the hydrophobic contribution of the poly- and perfluorinated carbon chain. Structurally distinct subclasses containing comparable fluorinated moieties, including ether-PFAS and fluorotelomer-derived compounds, generally exhibit chromatographic behavior that remains consistent with the same underlying retention mechanism. Thus, the RT–RI model is intended as an orthogonal plausibility filter to identify candidates with chromatographic behavior inconsistent with their proposed structures, rather than as a definitive predictor of retention for every PFAS subclass. While the curated suspect list can be directly reused across studies, the RT–RI filtering step is inherently method-specific and should be adapted to the chromatographic conditions of each dataset to avoid incorrect candidate exclusion. Several candidate features were filtered against the dynamic 95% confidence prediction interval (absolute ΔCI < 150 RI units) as their measured RT–RI relationship deviated significantly from model-expected values, thus ensuring a set of analytically coherent PFAS candidates (Dataset 1 = 30 candidates; Dataset 2 = 506 candidates) for an interpretative and balanced PFAS evidence across independent exposure studies. To further evaluate the influence of fragment support on candidate retention and identification level, a MatchFactor sensitivity analysis was performed by progressively increasing the acceptance threshold (Figure S4). The results show markedly different behaviors for the two confidence categories. No reduction in retained candidates was observed up to the adopted MatchFactor threshold (0.10). Increasing the threshold to 0.15 resulted in the complete removal of *geq3* candidates from Dataset 1 and reduced the retained candidate pool in Dataset 2 to 14.4% (73/506), while preserving all *leq2* candidates. At even more stringent thresholds (≥0.30), only the five *leq2* candidates remained in Dataset 2, whereas Dataset 1 retained all but one *leq2* structure only at a MatchFactor of 0.50. These results indicate that *leq2* candidates are supported by robust fragment evidence and remain stable across a broad range of MatchFactor thresholds. Conversely, the rapid decline of *geq3* candidates with increasingly stringent thresholds is consistent with their reliance on a limited set of recurrent PFAS substructural fragments rather than compound-specific fragmentation patterns. This behavior is expected and further supports the intended interpretation of the *geq3* tier as an exploratory, hypothesis-generating representation of the plausible PFAS chemical space, rather than a collection of confirmed compound identifications.

#### Wide-Scope PFAS Candidates Overview

The final PFAS candidate lists for both datasets are provided in the “*Supporting_Table*” folder (Supporting information). These tables are organized to facilitate interpretation of structural variability at the feature level, distinguishing between candidates assigned to the same detected feature (isomers; Δm/*z* ≤ 0.005 Da, ΔRT < 0.1 s) and candidates sharing the same precursor exact mass but differing in chromatographic behavior (isobars; Δm/*z* ≤ 0.005 Da, ΔRT > 0.1 s). This layout enables direct evaluation of whether tentative candidates reflect unique feature structures or ambiguity arising from structural overlap, thereby tracking and contextualizing candidate assignment uncertainty. Thus, multiple tentative structures associated with a single feature are intentionally retained within the repository to explicitly capture the structural uncertainty and diversity supported by the available evidence, thereby preserving chemically plausible hypotheses for future retrospective reevaluation rather than enforcing a single annotation. Results overview on both datasets is reported in Table 1. In Dataset 1, 43% (*n* = 6/14) of the former identified and candidate PFAS were also confirmed by the proposed SSA reanalysis, within *leq2* and *geq3* identification confidence.^48^ The “missing” cases are mainly due to low MS^2^ signal intensity, which restrained XIC-based feature confirmation (MatchFactor = 0) after filtering, representing a technical limitation of the proposed framework and other NTA workflows. This is also consistent with the previous study, in which such features lacking reported fragmentation patterns were nonetheless confirmed by a combination of targeted analysis and SSA using an in-house ion list (≃1,400 entries) focused primarily on AFFF-related PFAS, complemented by the NIST PFAS suspects providing only precursor and molecular formula matching. On top of that, some of the former identified structures were not included among suspects because they were not present in the starting OECD list (*n* = 1, Hexafluoro-1,2-propanediol). SSA identified 3 additional *leq2* structures in the reanalyzed data, namely perfluoroundecanoic acid, perfluorononanoic acid, and N-ethylperfluoro-1-octanesulfonamidoacetic acid. Together with the original and new *leq2* annotations, reanalysis contributed markedly to the increase of tentative (i.e., *geq3*) PFAS features, identifying 18 *geq3* unique structures, including 5 PFAS isomeric features (see next section). An even larger increase was observed for Dataset 2, where the original 21 identifications^49^ expanded to 506 candidates, comprising 5 *leq2* PFAS, 328 unique *geq3* structures, and 173 isomers. Among the *leq2* entries, only 3 overlap with the previously confirmed PFAS, whereas 10 of them, excluding branched isomers, fell among the *geq3* structures after reanalysis (total *n* = 13/16, ∼80%). The remaining PFAS confirmed in the previous study were not retained for the following reasons. *N*-Methylperfluorobutanesulfonamide was absent from the initial OECD list, which included only its N-oxide alkyl precursors. In contrast, perfluorohexanesulfonic acid and perfluorooctanoic acid were excluded during the filtering stage, as no diagnostic fragments were anchored after the SSA first stage. Notably, two additional *leq2* PFAS were newly identified in Dataset 2, namely 1-chloroperfluorooctanesulfonic acid and perfluorododecanesulfonic acid. The observed “before vs. after” trade-off highlights the strong dependence of SSA candidate selection on how the suspect list is constructed. A common limitation of all SSA-based approaches (including the proposed framework) is the experimental support embedded in the suspect list that directly influences which candidates can be retained or excluded during filtering. In particular, the availability of authentic standards and experimentally validated fragments determines the specificity of candidate matches. This dependence is particularly critical in the case of PFAS, as they share common fragmentation pathways that generate recurrent diagnostic ions (e.g., fragments 118.9 and 168.9), which are observed in multiple subclasses. Although these fragments confirm fluorinated structural motifs, they alone lack sufficient structural specificity to uniquely resolve precursor–fragment relationships (especially for DIA data). Accordingly, the proposed framework intentionally prioritizes analytical selectivity over sensitivity, retaining only candidates supported by multiple orthogonal lines of evidence and sufficient fragment evidence. While this conservative strategy minimizes unsupported tentative annotations, it also accepts that some genuine PFAS may not be retained when the archived HRMS data do not contain sufficient diagnostically informative MS^2^ evidence to support confident retrospective confirmation. Indeed, when only one or two characteristic fragments support a *geq3* annotation, the reported structure should be interpreted as a tentative, exploratory hypothesis rather than a confirmed compound identification. For this reason, introducing a relationship between structure and retention in the selection of candidate PFAS allows for attributing a complementary level of confidence to support a reduced or not very specific spectral match. Overall, most of the previously confirmed/provisional PFAS structures were captured by the proposed framework, also contributing to a broader interpretation of PFAS occurrence within the investigated HRMS datasets. Although the present workflow was developed and evaluated using DIA datasets, the proposed RT–RI and MS^2^ filtering strategy is also compatible with DDA acquisitions, which provide cleaner fragmentation patterns and reduce spectral ambiguity. Conversely, because DDA preferentially fragments the most abundant precursor ions, low-intensity PFAS features may remain without MS^2^ information, thereby limiting retrospective candidate confirmation. Consequently, the relative benefit of the present framework depends on the acquisition strategy employed. Under DIA conditions, the RT–RI model provides an important orthogonal constraint to increase confidence in fragment-supported annotations, whereas DDA conditions benefit from tools leveraging high-quality fragmentation patterns and homologous-series relationships (such as FluoroMatch).

**Table 1:** Number of PFAS Identifications Originally Obtained (Former Identifications) and Those Generated through Wide-Scope Suspect Screening Reanalysis, Categorized by Confidence Levels (*leq2*: Higher-Confidence/Confirmed Identifications; *geq3*: Tentative Identifications).^41^ ^a^

	Former Identifications		Wide-Scope SSA Reanalysis				
Dataset	leq2	geq3	leq2	geq3	Isomers	Total	Overlap
Dataset 1 ^48^	6	8	7	18	5	30	6/14
Dataset 2 ^49^	21	n.a.	5	328	173	506	13/16^b^

a For the geq3 group, SSA results are separated into unique structures and isomeric assignments. Totals refer to the wide-scope SSA results (Dataset 1: *n* = 30, Dataset 2: *n* = 506). Overlaps indicate the former PFAS confirmed by the proposed framework. n.a. = not applicable. The complete list of SSA candidates can be found in Supplementary Tables.

b Branched isomers (*n* = 5) excluded.

#### PFAS Trends in Reanalyzed Datasets

The proposed framework enables the joint evaluation of structural-chromatographic behavior (*m*/*z*, RI), mass-defect patterns, and MS^2^ similarity, revealing a chemically coherent PFAS suspect set (Figure 3). Regarding Dataset 1, in the RI–*m*/*z* space (Figure 3A), suspect candidates follow a monotonic increase in RI with increasing *m*/*z*, reflecting a coherent drift of perfluorinated chain length on reversed-phase retention. Two mass windows contain features with equal RT but slightly different RI values (ΔRI < 100), indicative of tentative isomeric PFAS species differing in functionalization or branching, yet identical or closely related molecular formulas leading to comparable chromatographic behavior. Figures S5 and S6 show the tentative isomeric structures compatible (RI and MS^1^-MS^2^) with these features (i.e., sulfate-sulfonate ethers and unsaturated alcohols/aldehydes/ethers), further highlighting the potential chemical variability that occurs in PFAS space that the presented wide-scope framework can map. Kendrick mass defect analysis (KMD, CF_2_ normalization; Figure 3B) further confirms the PFAS-specific nature of these candidates. A large fraction of suspects aligns along well-defined homologous series (e.g., KMD = 0.03–0.04: sulfonic acid derivatives; KMD = 0.005–0.01: carboxylic acids; KMD = −0.01–0.02: sulfonamides) characterized by regular CF_2_ mass increments, consistent with perfluoroalkyl chain elongation. Candidates assigned to these homologous series included the majority of the confirmed/tentative PFAS occurring in Dataset 1, also comprising the highest MatchFactor entries (*leq2*). Even though less representative, isolated singletons suggest the potential occurrence of structurally distinct PFAS subclasses (e.g., fluorotelomer amines and sulfonamides), thus eventually suggesting a more complex PFAS exposure that is generally overlooked. In Dataset 2, the wide-scope SSA revealed a substantially more complex and diverse PFAS exposure profile. The distribution of unique mass windows in the RI–*m*/*z* space (*leq2* = 5 and *geq3* = 328) follows a monotonic retention trend (Figure 3C) comparable to that observed for Dataset 1, indicating the presence of two pairs of tentative isobar entries (Table S3), several isomeric structures, and multiple PFAS homologous series, as also confirmed in KMD distribution (Figure 3D). Within the sulfonate–sulfate homologous series (KMD = 0.038–0.043), *leq2*-confirmed PFBS and PFDoDS provide high-confidence anchors that validate the presence of this PFAS class across Dataset 2. At the same time, this series exposes isomeric complexity, as illustrated by the feature at *m*/*z* = 514.9249, for which two sulfonate–sulfate derivatives are tentatively assigned (Figure S7). Similarly, other homologous families, including perfluoroalkanes (KMD = −0.0003 to −0.0004; Figure S8) and pyrimidine- and imidazole-substituted PFAS (KMD = −0.0241 to −0.0243; Figure S9), exhibit multiple isomeric candidates sharing the same precursor mass and MS^2^ fragmentation. These results demonstrate that the wide-scope SSA reanalysis moves by far beyond target-PFAS detection to a chemical space-based interpretation of PFAS exposure, capturing confirmed and tentative homologous series, possible isomeric clusters, and structurally distinct subclasses. Despite the two datasets representing different exposure circumstances (i.e., direct occupational and indirect exposure in Dataset 1, population-level exposure in Dataset 2), to contextualize the implications of the expanded PFAS detection in terms of exposure processes, we evaluated cross-study patterns at the level of PFAS chemical space. The wide-scope reanalysis highlighted four PFAS homologous series shared across both datasets (Figure 4 and “*Supporting_Table*”), including tentative subclasses that are typically underrepresented in conventional workflows and possibly overlooked by former studies, such as keto-fluorotelomer alcohols (K-FTTh-EtOHs) and ethyl perfluorosulfonamidoacetic acids (EtFOSAAs). These homologous patterns, identified within narrow KMD windows (0.005 < KMD < 0.01) and supported by consistent mass–retention behavior, suggest that structurally related PFAS may be present across distinct exposure scenarios. While some of these observations remain tentative, the occurrence of the confirmed homologous series (e.g., PFASAs) across datasets with different exposure contexts may point toward overlapping contributions from both localized (i.e., occupational) and more diffuse environmental or consumer-related sources. In this sense, the observed overlap is interpreted as indicative of broader PFAS chemical space patterns rather than definitive evidence of specific exposure pathways. At the same time, the proposed framework remains inherently dependent on the composition of the suspect list, the quality and specificity of the available MS^2^ information, and the chromatographic applicability domain of the investigated datasets. Despite these limitations, the reanalysis framework confidently expanded the reported PFAS profile through the recovery of additional confirmed (*leq2*) homologs, while broadening the exploratory PFAS chemical space via *geq3* candidate series that warrant future confirmation. We believe that continuous updating of suspect lists, spectral resources, and retention-behavior models provides more adaptive and chemically informed PFAS exposure assessment in exposomics studies.

**Figure 3:**
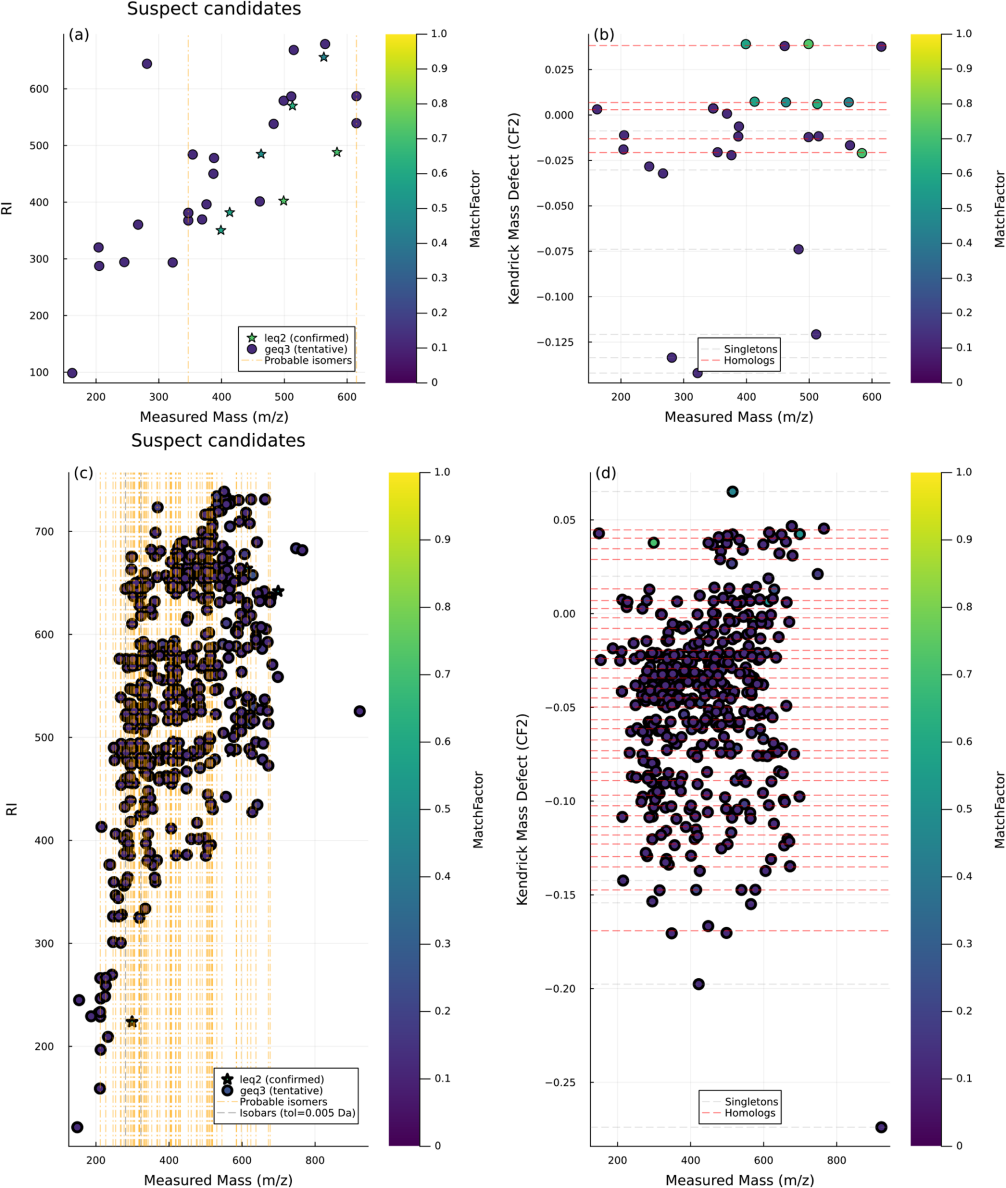
Visualization of suspect candidates from Datasets 1 (a–b) and 2 (c–d) based on mass, retention, and mass-defect criteria. (a–c) Measured mass (*m*/*z*) vs retention index (RI) for *geq3* tentative compounds (circles) and *leq2* confirmed with standards (stars); dashed vertical lines indicate regions of probable isomeric candidates and isobars. (b–d) Kendrick mass defect (CF_2_-based) vs *m*/*z*, highlighting homologous series (red dashed lines) and singleton features. Point color represents the MS^2^ MatchFactor.

**Figure 4:**
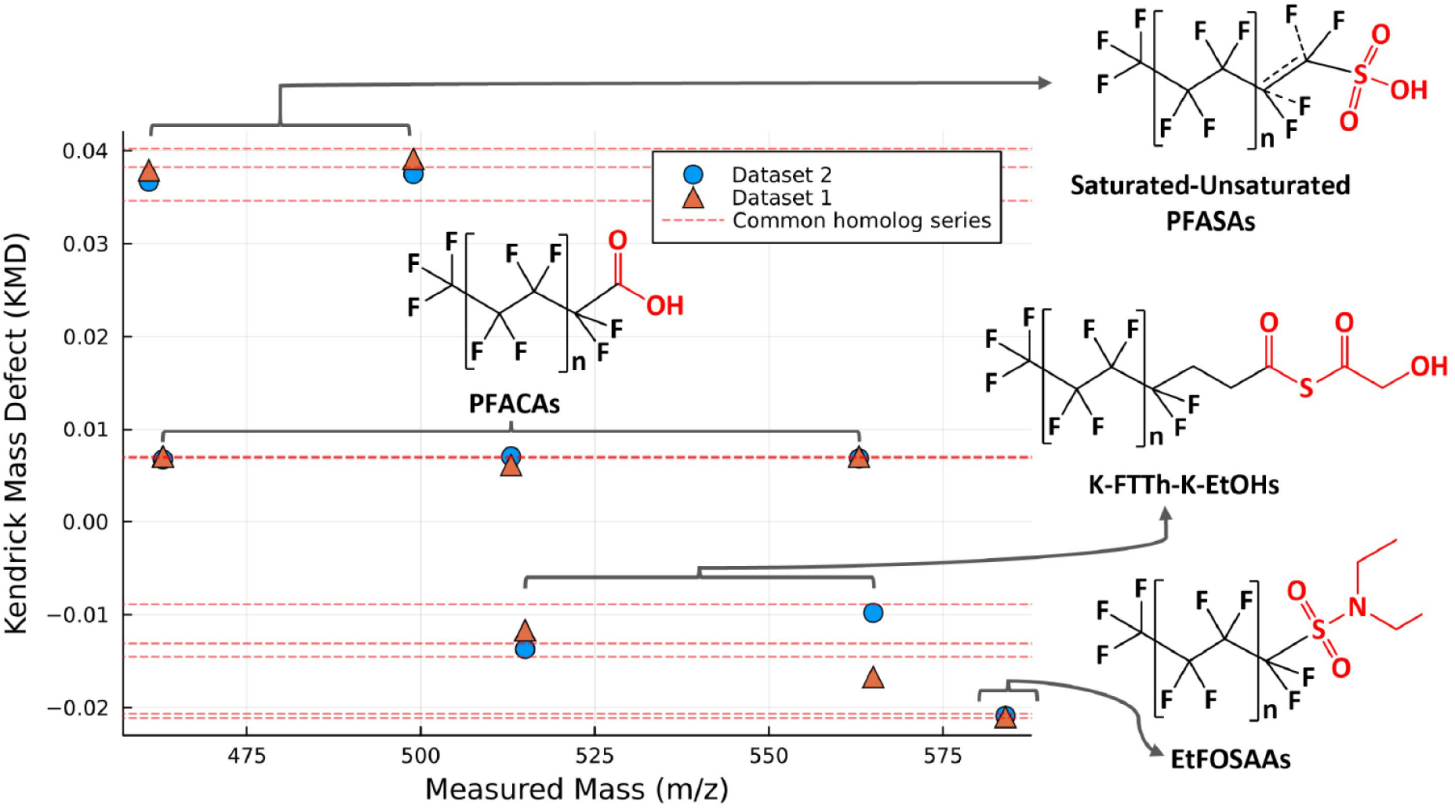
Kendrick mass defect (KMD) plot showing shared PFAS homologous series across Datasets 1 and 2. Red dashed lines indicate common homologue series regions detected within narrow windows of 0.005 < KMD < 0.01. Homologous series acronyms: PFASAs = perfluoroalkyl sulfonic acids; PFACAs = perfluoroalkyl carboxylic acids; K-FTTh-K-EtOH = keto-fluorotelomer alcohols; EtFOSAAs = ethyl perfluoro sulfonamidoacetic acids.

### Retrospective Evidence on PFAS Exposure: Continuous Reporting

Given the vast diversity of PFAS within the human and environmental exposome, only a small fraction currently has broad experimental confirmation. At the same time, numerous tentatively identified PFAS detected by the community in human and environmental samples provide valuable insights into the broader exposure landscape. However, the utility of these tentative findings remains limited by the lack of structured, community-level mechanisms for their systematic collection, updating, and reinterpretation. In this context, we propose a community-oriented repository of measured and tentatively identified PFAS in exposomics studies. The objective is not only to improve transparency and reproducibility but also to enable continuous knowledge integration through retrospective reanalysis. As an initial step, we provide public access to PFAS exposure signatures derived from both datasets herein investigated: https://gitlab.com/lrenai/PFAS_Exposure_Repo.git. This “first-attempt” repository contains individual structure-centric records for each detected PFAS suspect, including confirmed and tentatively assigned candidates, formatted to support cross-study comparison and reannotation (Figure S10). Each repository entry includes standardized molecular descriptors and mass spectrometric and chromatographic data fields designed to preserve structural interpretability while maintaining sample anonymity (see also section Retrospective Repository of PFAS Candidates). The information architecture was intentionally structured to facilitate interoperability with community spectral resources and annotation platforms, enabling future integration into reference databases such as MassBank. This resource is designed to support both independent screening of external datasets using method-contextualized PFAS candidates and iterative structural confirmation as new analytical standards, spectral data, or suspect entries become available. A clear example is provided by Dataset 1. As discussed earlier, some PFAS reported in the original study were not captured in the first round of reanalysis because they were absent in the initial OECD-based suspect list used in the first screening step. Reprocessing the dataset with an updated suspect list enabled the retrieval of the previously identified hexafluoropropanediol isomer structures. As an alternative case, we confirmed the occurrence of a PFAS tentative structure that fell outside the RPLC classification, i.e., tris­(2-methylpropyl) 3-(perfluorobutanamido)­propane-1,1,3-tricarboxylate. This demonstrates the gain in chemical knowledge when new or updated candidates are incorporated in the framework of sample reanalysis (see Supplementary_Tables). This example highlights how continuous updating of suspect repositories can improve the interpretability of PFAS occurrence in human samples and support a more dynamic characterization of exposure. By enabling the incorporation of newly reported compounds and structural hypotheses, such repositories facilitate the identification of emerging PFAS signals that may otherwise remain unrecognized in retrospective datasets. In this context, continuous reporting supports the transition from static, target-limited monitoring toward a more adaptive exposure assessment framework, where both confirmed and tentative PFAS features contribute to identifying relevant chemical patterns, prioritizing compounds for follow-up analysis, and informing future biomonitoring strategies. Such discrepancies illustrate the evolving nature of PFAS knowledge and reinforce the importance of iterative reanalysis as a mechanism to progressively refine exposure characterization, particularly for structurally diverse and underrepresented PFAS subclasses.

## Supplementary Material



## Notes

Details and results of the retrospective suspect screening workflow (Sections S1–S5) (PDF)
