# Supplementary material for: Understanding and Reporting PFAS Exposure: Wide-Scope Retrospective Suspect Screening of High-Resolution Mass Spectrometry Data from Exposomics Studies
Source: Environ Sci Technol. 2026 Jul 22;60(30):20790–800. doi: 10.1021/acs.est.6c03864 (PMC13449885; doi:10.1021/acs.est.6c03864)
Supplement: Supplementary file 1 [file es6c03864_si_001.pdf]

Supporting information for: "Understanding and Reporting PFAS Exposure: Wide-scope Retrospective Suspect Screening of High-resolution Mass Spectrometry Data from Exposomics Studies"

Lapo Renai,[*,†,‖] Federica Calabrò,[†] Viktoriia Turkina,[†] Pradeep Dewapriya,[‡] Kevin V. Thomas,[‡] Stefano Papazian,[¶] and Saer Samanipour[*,†,§,‡]

†*Van 't Hoff Institute for Molecular Sciences (HIMS), University of Amsterdam, 1090 GD, Amsterdam, the Netherlands*

‡*Queensland Alliance for Environmental Health Sciences (QAEHS), 20 Cornwall Street, Woolloongabba, QLD, 4102, Australia*

¶*National Facility for Exposomics, Science for Life Laboratory, Stockholm University, Solna 171 65, Sweden*

§*UvA Data Science Center, University of Amsterdam, Amsterdam*

‖*Corresponding author*

E-mail: l.renai@uva.nl; s.samanipour@uva.nl

The *Supporting information* has 15 pages and includes supplementary details about the proposed framework and analyzed data, supported also by 3 tables (Tables S1-S3) and 10 figures (Figures S1-S10).

List of Figures

S1 Distribution of Kenrick mass defects (KMD, CF2-based), retention indices (RI, UoA scale), and exact masses for the ≃ 4900 OECD PFAS structures according to the RPLC classification, “inside”=1, “maybe”=0, and “outside”=-1 the reversed phase domain. S5

S2 Representative examples of MS^1–MS^2 matching outcomes within the suspect screening workflow. (A) Positive match: a well-defined MS^1 chromatographic peak is associated with coherent MS^2 fragmentation patterns within the retention time window, supporting fragment-to-precursor consistency. (B) Negative match: although a distinct MS^1 signal is observed, the corresponding MS^2 signal lacks structured fragmentation and is dominated by non-specific or background contributions. S7

S3 Filtering strategy workflow implemented in the suspect screening reanalysis of Dataset 1 and Dataset 2. . . . . S8

S4 Percentage of retained candidates within each confidence level (*leq2* and *geq3*) vs MatchFactor threshold. Percentages were calculated independently for each confidence level and dataset. S9

S5 Dataset 1 - m/z-RI distribution in the sample of suspect PFAS isomers @m/z=614.9231, related extracted ion chromatogram as well as MS^1 and MS^2 data. S10

S6 Dataset 1 - m/z-RI distribution in the sample of suspect PFAS isomers @m/z=346.9742, related extracted ion chromatogram as well as MS^1 and MS^2 data. S11

S7 Dataset 2 - m/z-RI distribution in the sample of suspect PFAS isomers @m/z=514.9249, related extracted ion chromatogram as well as MS^1 and MS^2 data. S12

S8 Dataset 2 - m/z-RI distribution in the sample of suspect PFAS isomers @m/z=318.9799, related extracted ion chromatogram as well as MS^1 and MS^2 data. S13

S9 Dataset 2 - m/z-RI distribution in the sample of suspect PFAS isomers @m/z=247.0083, related extracted ion chromatogram as well as MS^1 and MS^2 data. S14

S10 Example of a PFAS repository entry with available metadata and structural-experimental records. S16

List of Tables

S1 Summary of the LC–HRMS acquisition parameters for the two datasets. DIA = data-independent acquisition; N.A. = not applicable. S4

S2 Examples of illogical PFAS candidates retrieved after the application of the chemically informative suspect list and before data- and model-based filtering. S8

S3 Tentative isobaric clusters detected in Dataset 2. Each row represents a group of features sharing the same measured mass within tolerance but exhibiting different retention times and InChIKeys. S11

S1. Instrumental conditions & Curated PFAS Suspect List

Table S1: Summary of the LC–HRMS acquisition parameters for the two datasets. DIA = data-independent acquisition; N.A. = not applicable.

**Parameter**	**Dataset 1**	**Dataset 2**
Study	General and occupation-exposure populations (n = 107)	Healthy and chronic-disease population (n = 161)
Sample	Serum pools	Plasma
Extraction	N.A.	HybridSPE-Phospholipid cartridges
LC	UHPLC C18	UHPLC C18
MS analyzer	Q-TOF	QE Orbitrap
Acquisition mode	SWATH-DIA	DIA
Precursor mass range	1001100 Da	90–1000 Da
Isolation window	50 Da	237 Da
Window overlap	1 Da	10 Da
MS^1 resolution	25,000	120,000
MS^2 resolution	25,000	30,000

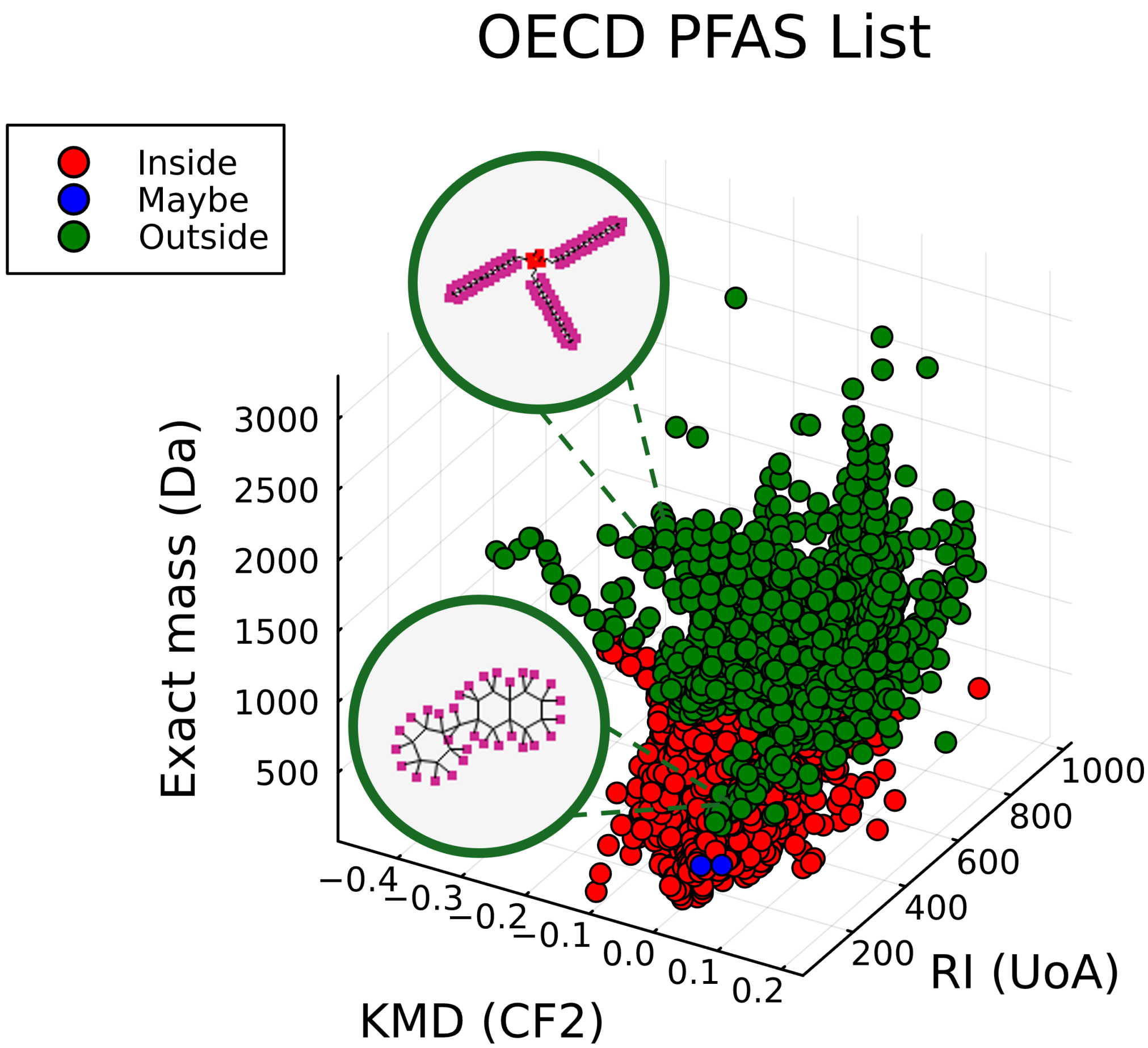

Figure S1: Distribution of Kenrick mass defects (KMD, CF2-based), retention indices (RI, UoA scale), and exact masses for the ≃ 4900 OECD PFAS structures according to the RPLC classification, “inside”=1, “maybe”=0, and “outside”=-1 the reversed phase domain.

S2. Suspect Screening algorithm

For each suspect entry, an extracted ion chromatogram (XIC) is generated from MS^1 data using the theoretical precursor mass and the defined mass tolerance. Candidate chromatographic peaks are detected either directly from the XIC using the minimum intensity threshold. Peaks lacking a detectable chromatographic maximum were discarded. For each retained peak, the corresponding MS^1 spectrum at the chromatographic apex is evaluated for isotopic consistency with the theoretical precursor mass. Only peaks showing a compatible isotope

pattern within the specified mass tolerance are retained. A retention-time window around the MS^1 peak apex is then used to retrieve MS^2 spectra compatible with the candidate precursor (Figure S2). If no diagnostic MS^2 fragment ions can be extracted above the noise level within the defined mass tolerance and retention-time window, the candidate is retained only with MS^1 and isotopic evidence and assigned no fragment support (filtered later in the framework). Otherwise, the expected MS^2 ions are matched to the experimentally extracted fragment ions within the defined mass tolerance. Also, XIC consistency (retention window and intensity) between precursor and fragments is checked for a positive match (Figure S2). Matched fragment intensities are normalized and square-root transformed before comparison with the reference fragment intensity vector to calculate the MatchFactor. Therefore, the MatchFactor represents a weighted fragment agreement score, while precursor mass accuracy, isotope pattern, retention time, and chromatographic coherence provide additional orthogonal evidence.

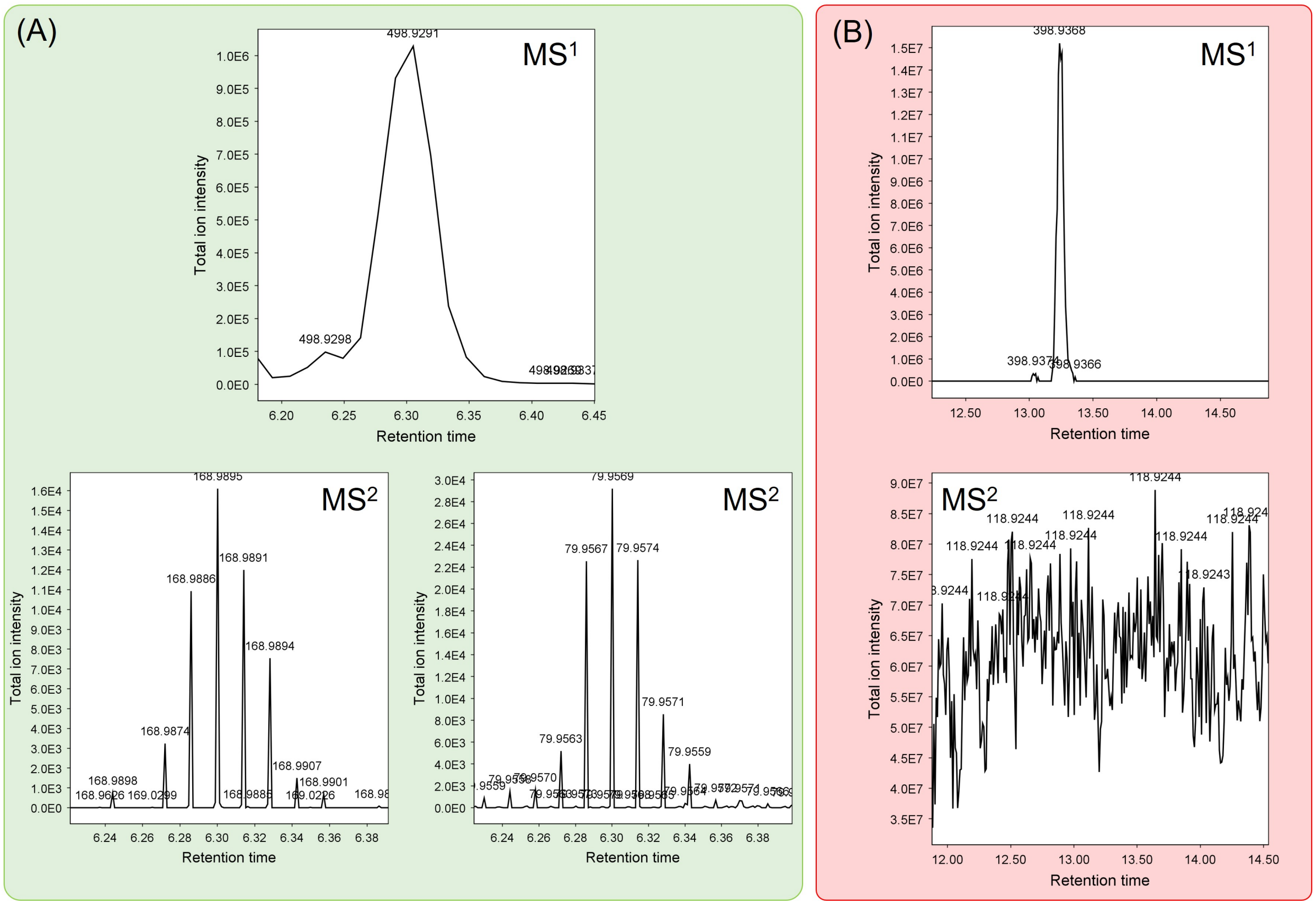

Figure S2: Representative examples of MS^1–MS^2 matching outcomes within the suspect screening workflow. (A) Positive match: a well-defined MS^1 chromatographic peak is associated with coherent MS^2 fragmentation patterns within the retention time window, supporting fragment-to-precursor consistency. (B) Negative match: although a distinct MS^1 signal is observed, the corresponding MS^2 signal lacks structured fragmentation and is dominated by non-specific or background contributions.

$$\text{MatchFactor} = \text{round}\left(\sum_{i=1}^{n} f_i \cdot \sqrt{\frac{I_i}{\sum_{j=1}^{n} I_j}}\right) \tag{1}$$

where f_i represents the presence or weighting of fragment i in the candidate structure, and I_i corresponds to the measured intensity of fragment i in the MS^2 spectrum.

S3. SSA Filtering Strategy

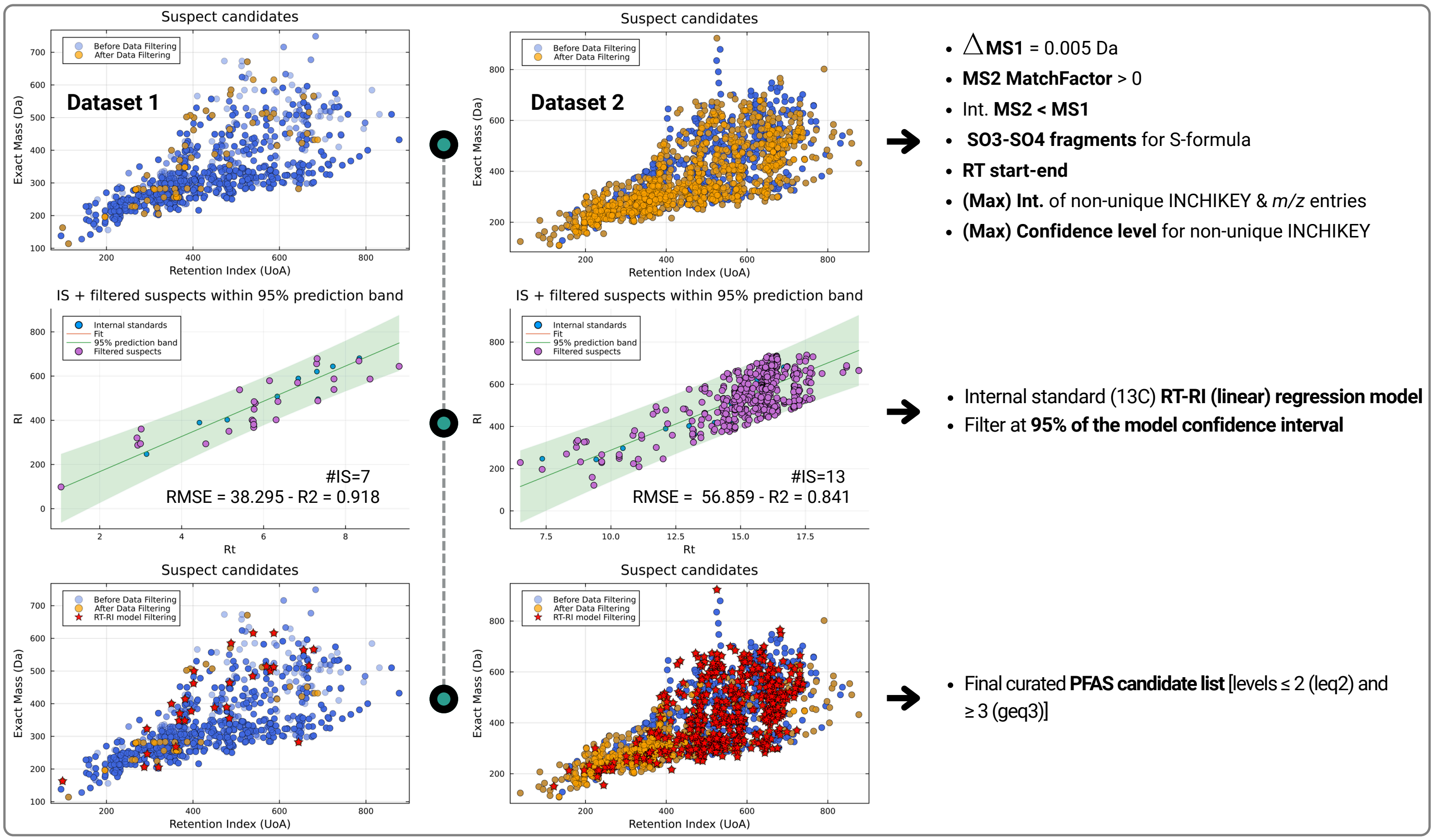

Figure S3: Filtering strategy workflow implemented in the suspect screening reanalysis of Dataset 1 and Dataset 2.

Table S2: Examples of illogical PFAS candidates retrieved after the application of the chemically informative suspect list and before data- and model-based filtering.

Formula	m/z	MS^1 Int.	MS^2 frags.	MS^2 Int.	InChIKeys
C5H6F7N	212.0305	2368	79.9574, 80.9652	29592, 8043	VYXXEPNGYRIBCL-UHFFFAOYSA-N
C8H16F4N	201.1133	8378	79.9574	10095	ZKQUIOUGNRZWOL-UHFFFAOYSA-N

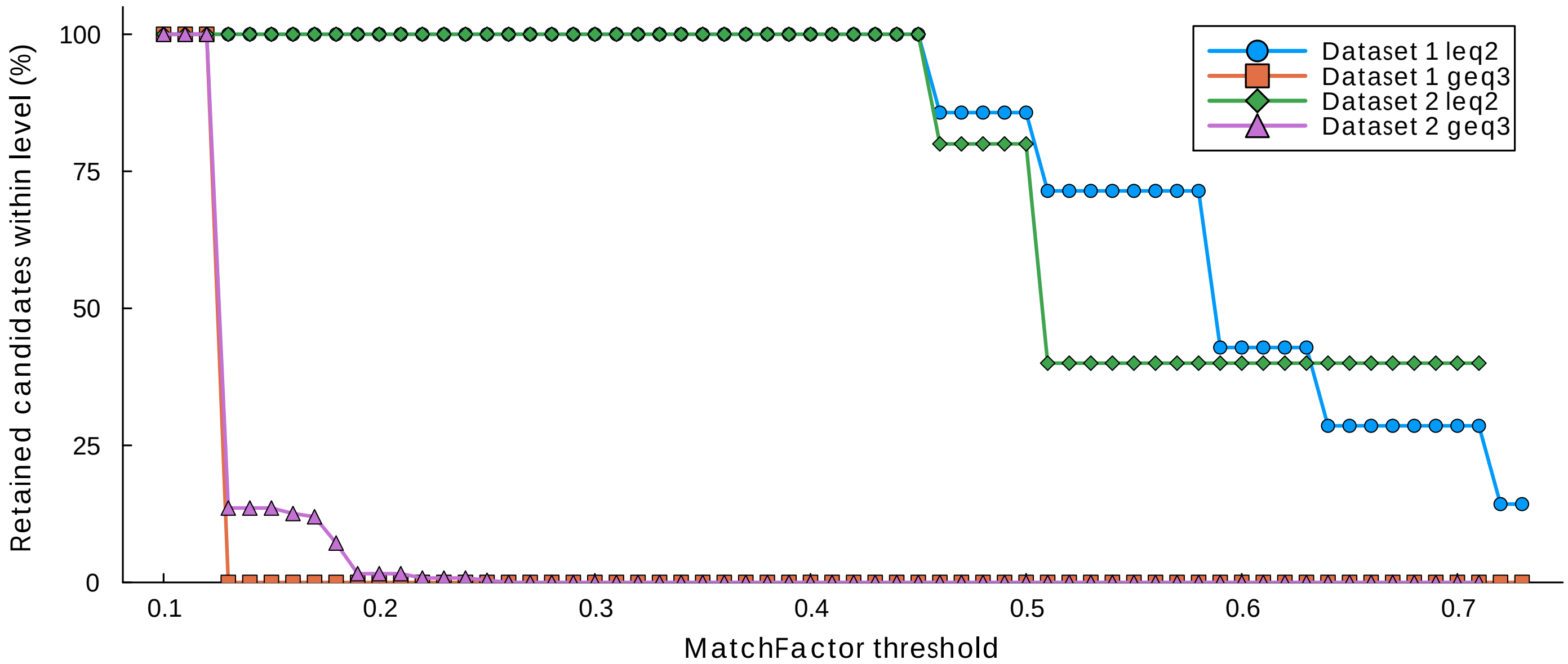

Figure S4: Percentage of retained candidates within each confidence level (*leq2* and *geq3*) vs MatchFactor threshold. Percentages were calculated independently for each confidence level and dataset.

S4. Reanalysis results

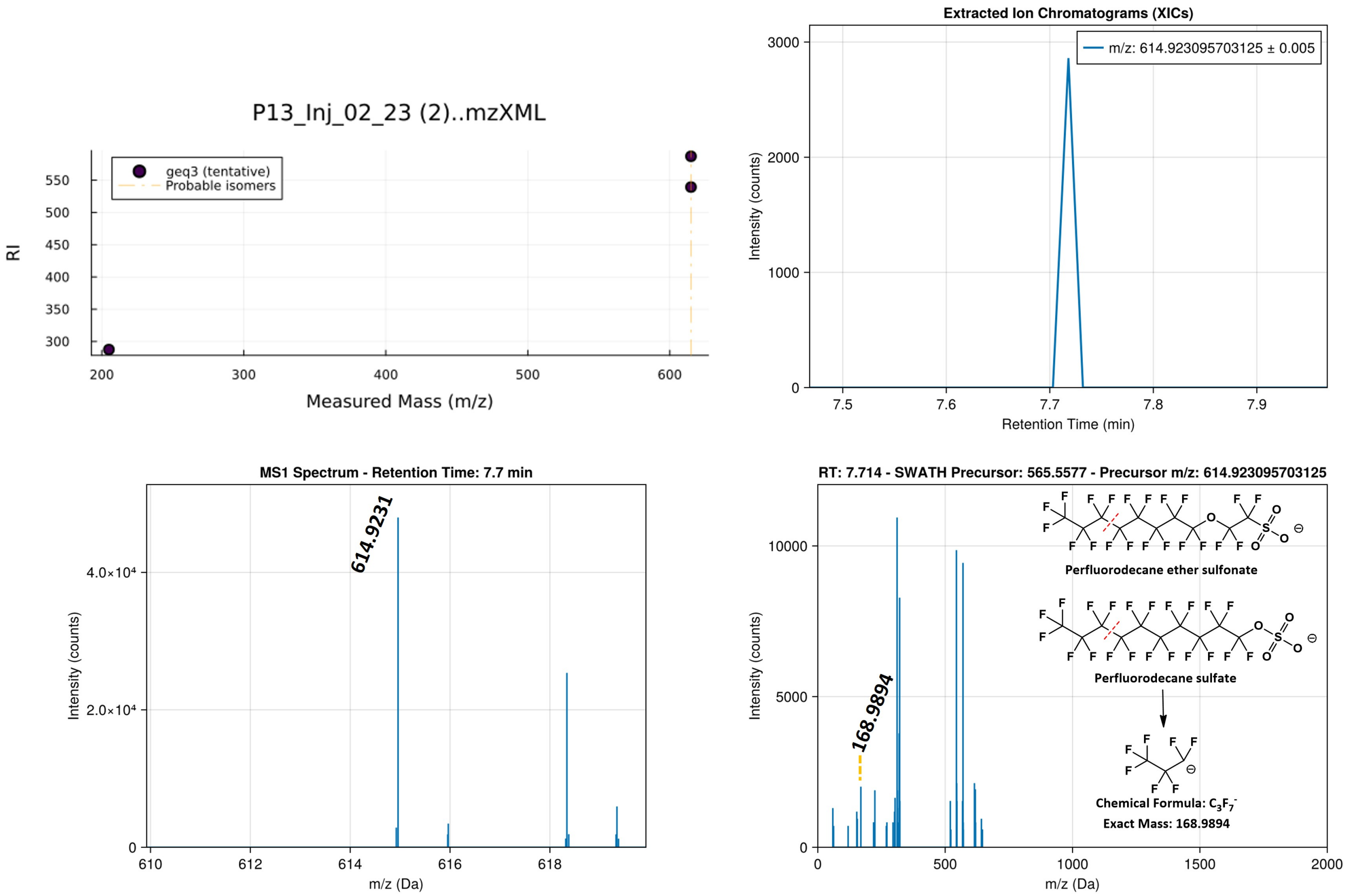

Figure S5: Dataset 1 - m/z-RI distribution in the sample of suspect PFAS isomers @m/z=614.9231, related extracted ion chromatogram as well as MS^1 and MS^2 data.

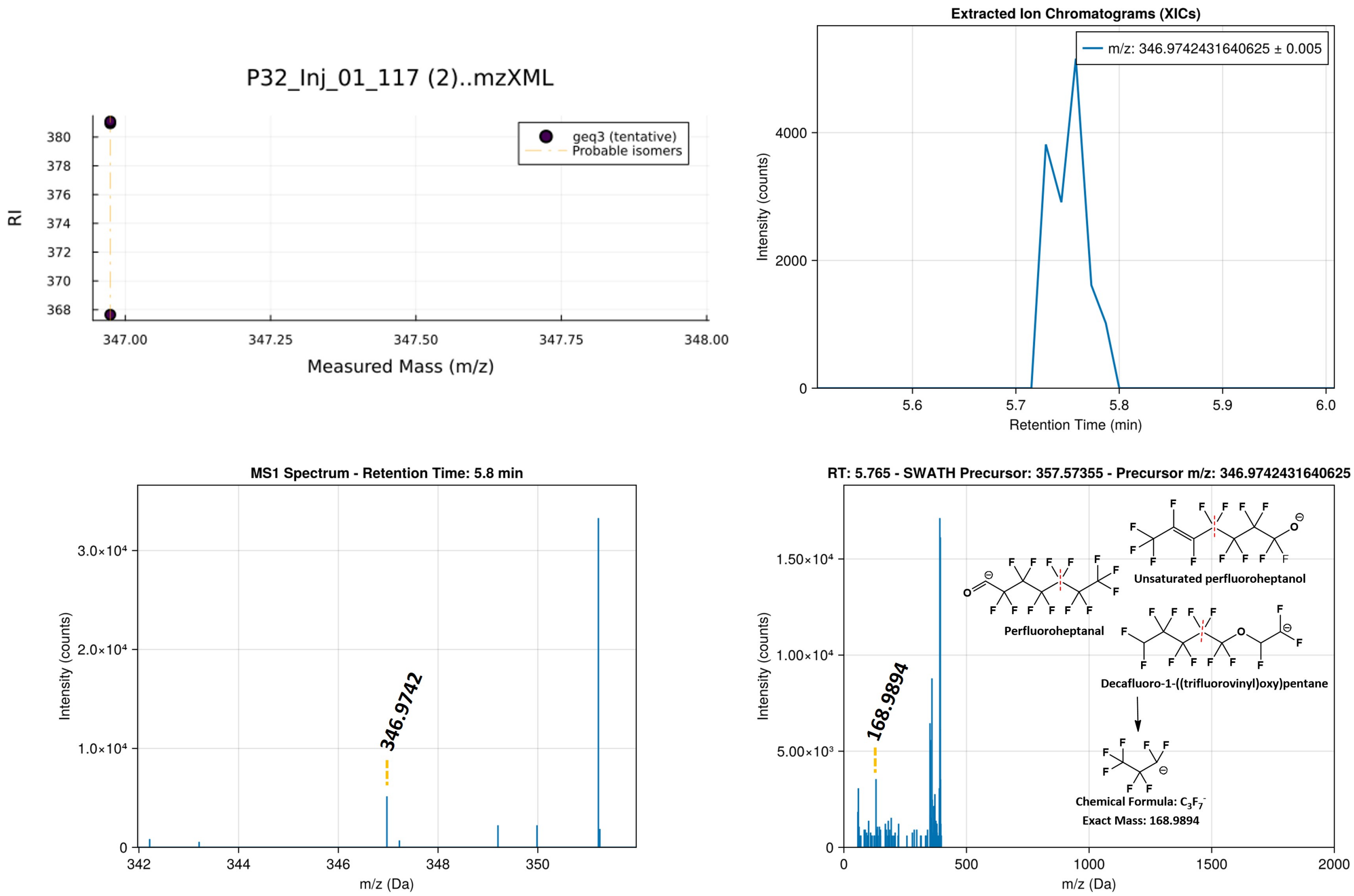

Figure S6: Dataset 1 - m/z-RI distribution in the sample of suspect PFAS isomers @m/z=346.9742, related extracted ion chromatogram as well as MS^1 and MS^2 data.

Table S3: Tentative isobaric clusters detected in Dataset 2. Each row represents a group of features sharing the same measured mass within tolerance but exhibiting different retention times and InChIKeys.

m/z center	RT min	RT max	RT span	Entries	InChIKeys
281.0475	12.368	17.088	4.720	2	VZOWCSFBTSNJKX-UHFFFAOYSA-N<br>NEIFNBMQYNENQZ-UHFFFAOYSA-N
323.0099	15.908	15.937	0.029	2	FVBNTOULJJXFBP-UHFFFAOYSA-N<br>BZEGSOOPSLUPSS-UHFFFAOYSA-N

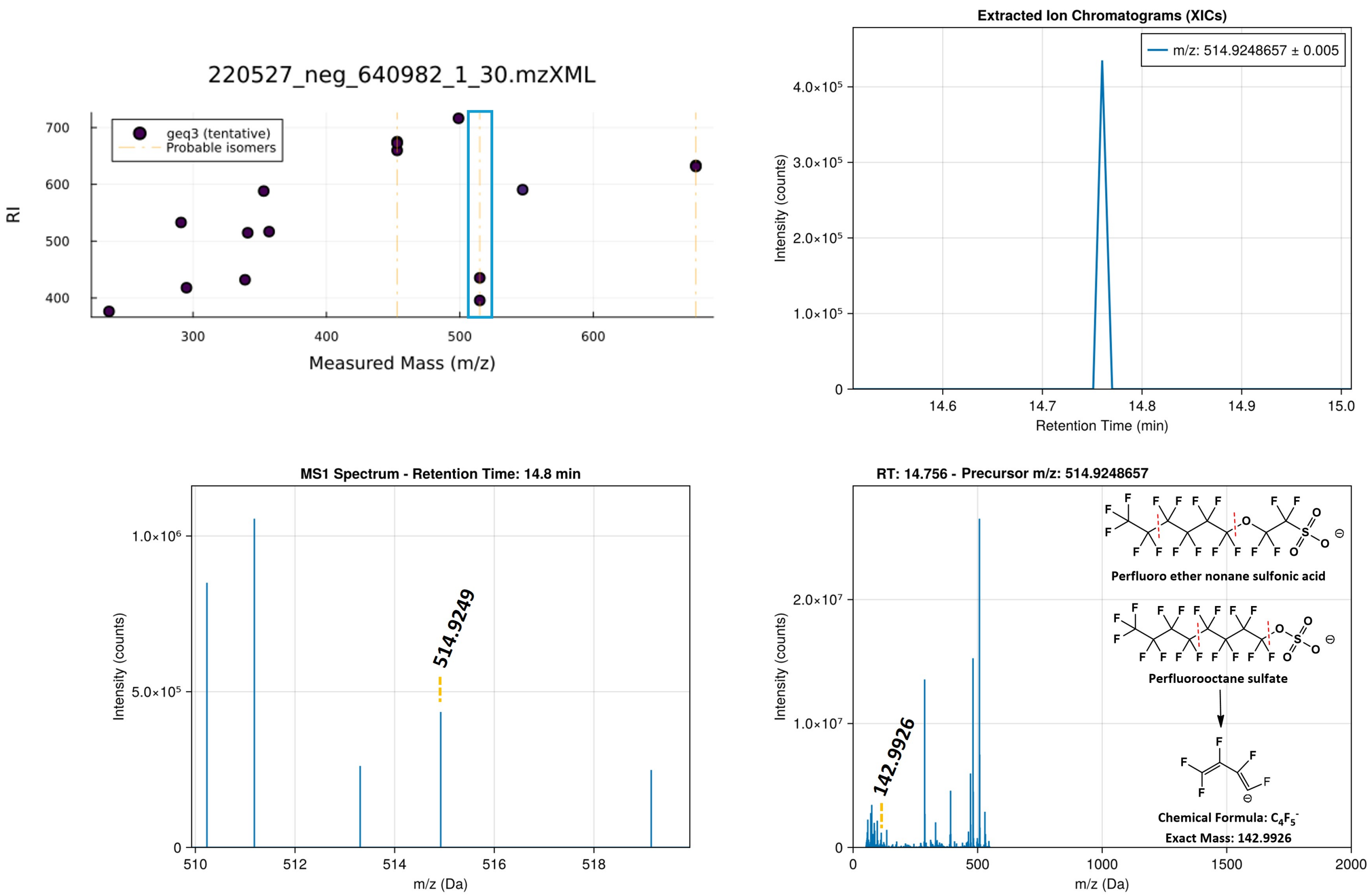

Figure S7: Dataset 2 - m/z-RI distribution in the sample of suspect PFAS isomers @m/z=514.9249, related extracted ion chromatogram as well as MS^1 and MS^2 data.

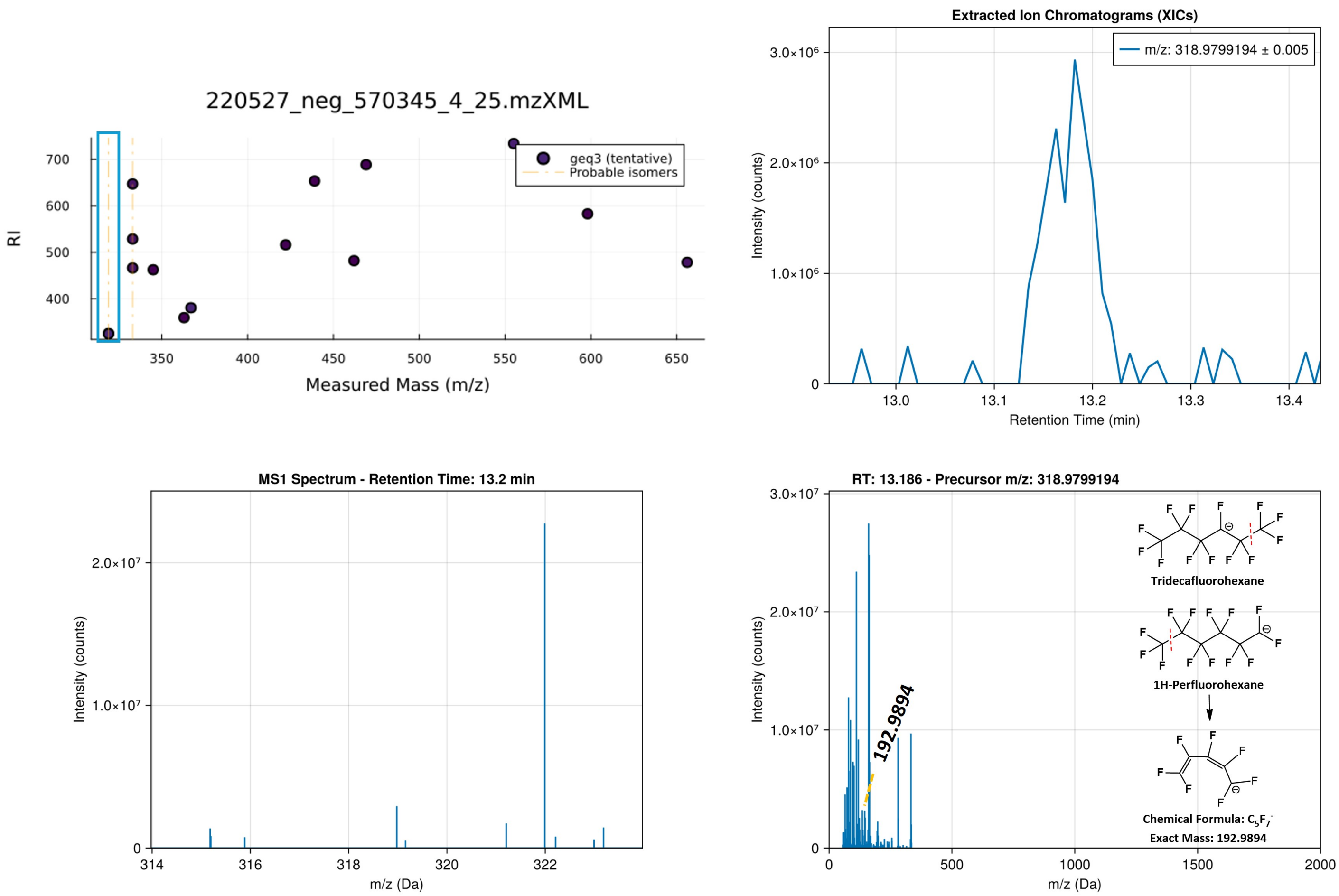

Figure S8: Dataset 2 - m/z-RI distribution in the sample of suspect PFAS isomers @m/z=318.9799, related extracted ion chromatogram as well as MS[1] and MS[2] data.

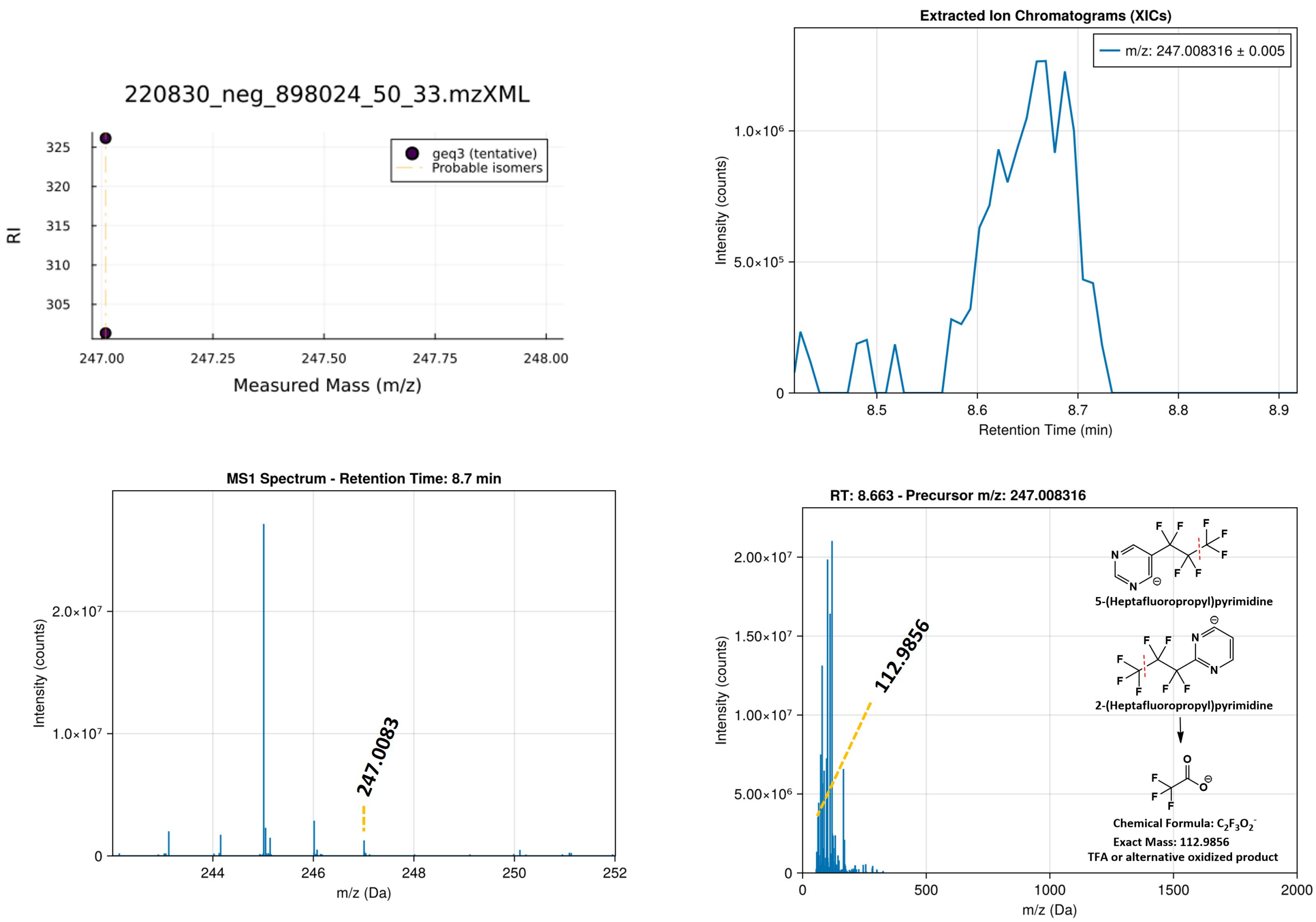

Figure S9: Dataset 2 - m/z-RI distribution in the sample of suspect PFAS isomers @m/z=247.0083, related extracted ion chromatogram as well as MS^1 and MS^2 data.

S5. Pipeline for retrospective PFAS repository

To improve transparency, reuse, and long-term interpretability of wide-scope PFAS suspect screening outputs, we compiled all curated suspect candidates into a structure-centric repository. Each candidate is stored as an individual text record (.txt) formatted in a MassBank-inspired style. Records include chemical identifiers and experimental evidence required for later confirmation and cross-study comparison, while excluding sample identifiers to protect privacy and prevent tracing to individual files or participants. Column-name normalization was applied to standardize headers across datasets' results as *.csv* in-

puts. Confidence reporting was mapped to ensure consistent interpretation across datasets as follows: "≤ 2" - candidate structures confirmed MS^2 data from standards and/or experimental results from literature; "≥ 3" - tentative candidate structure consistent with structure (MS^2 components and retention consistency). To prevent linkage back to samples or individuals, source-specific fields were excluded, including file- and sample-traceable identifiers (e.g., raw filename, sample ID, dataset labels). Each entry was assigned a sequential anonymous accession identifier of the form: PFAS000001, PFAS000002, ... (one accession per row of the curated suspect tables). No encoding of dataset origin, sample origin, or study metadata was included in the accession. Field prefixes were used to distinguish chemical descriptors from experimental metadata, following a MassBank-inspired convention: "CH$" fields - chemical/structural descriptors (e.g., CH$Name, CH$Formula, CH$INCHIKEY, CH$EXACT_MASS, CH$Identification_level); "PK$" fields - experimental and peak-related metadata (e.g., PK$Rt, PK$MeasMass, PK$Int, PK$MatchFactor, PK$RI, PK$KMD). Acquisition and chromatography descriptors were included as dedicated metadata lines to support interpretability: AC$MASS_SPECTROMETRY documenting acquisition strategy (e.g., DDA or DIA), AC$CHROMATOGRAPHY documenting chromatographic selectivity (e.g., RPLC). These fields were assigned based on the processed *.csv* source, but without including any dataset name. MS^2 fragment evidence was exported as a tabular peak list.

```
ACCESSION: PFAS000001
RECORD_TITLE: PFAS suspect entry
DATE: 2026-02-03
COMMENT: Generated from curated suspect-screening results; anonymous record ID.
AC$MASS_SPECTROMETRY: HRMS/MS; SWATH
AC$CHROMATOGRAPHY: RPLC
REFERENCE:DOI: https://doi.org/10.1021/acs.est.3c00663
CH$Name: Perfluorooctanesulfonic acid (PFOS)
CH$Formula: C8HF17O3S
CH$INCHIKEY: YFSUTJLHUFNCNZ-UHFFFAOYSA-N
CH$EXACT_MASS: 499.9375
CH$Identification_level: <=2
CH$KMD: 0.0391
CH$RI: 402.31476
PK$Nr: 147
PK$Rt: 6.305
PK$MeasMass: 498.9290771
PK$Int: 1.03e6
PK$IsoDepth: 1
PK$Isotop: [499.93328857421875]
PK$NrMatchedFrags: 2
PK$NrTestedFrags: 3
PK$MatchFactor: 0.73
PK$SpectraType: EXP
PK$RI_pred: 510.4732
PK$PEAK: m/z int.
118.9922 3018.0000
79.9572 28145.0000
PK$N_PEAK: 2
```

Figure S10: Example of a PFAS repository entry with available metadata and structural-experimental records.
